# Combined Effect of Free Nitrous Acid Pretreatment and Sodium Dodecylbenzene Sulfonate on Short-Chain Fatty Acid Production from Waste Activated Sludge

**DOI:** 10.1038/srep21622

**Published:** 2016-02-12

**Authors:** Jianwei Zhao, Yiwen Liu, Bingjie Ni, Qilin Wang, Dongbo Wang, Qi Yang, Yingjie Sun, Guangming Zeng, Xiaoming Li

**Affiliations:** 1 College of Environmental Science and Engineering, Hunan University, Changsha 410082, P.R. China; 2 Key Laboratory of Environmental Biology and Pollution Control (Hunan University), Ministry of Education, Changsha 410082, P.R. China; 3 Advanced Water Management Centre, The University of Queensland, QLD 4072, Australia; 4 School of Environment and Municipal Engineering, Qingdao Technological University, Qingdao 266033, P.R. China

## Abstract

Free nitrous acid (FNA) serving as a pretreatment is an effective approach to accelerate sludge disintegration. Also, sodium dodecylbenzene sulfonate (SDBS), a type of surfactants, has been determined at significant levels in sewage sludge, which thereby affects the characteristics of sludge. Both FNA pretreatment and sludge SDBS levels can affect short-chain fatty acid (SCFA) generation from sludge anaerobic fermentation. To date, however, the combined effect of FNA pretreatment and SDBS presence on SCFA production as well as the corresponding mechanisms have never been documented. This work therefore aims to provide such support. Experimental results showed that the combination of FNA and SDBS treatment not only improved SCFA accumulation but also shortened the fermentation time. The maximal SCFA accumulation of 334.5 mg chemical oxygen demand (COD)/g volatile suspended solids (VSS) was achieved at 1.54 mg FNA/L treatment and 0.02 g/g dry sludge, which was respectively 1.79-fold and 1.41-fold of that from FNA treatment and sludge containing SDBS alone. Mechanism investigations revealed that the combined FNA pretreatment and SDBS accelerated solubilization, hydrolysis, and acidification steps but inhibited the methanogenesis. All those observations were in agreement with SCFA enhancement.

Recently, biological production of short-chain fatty acid (SCFA) from waste activated sludge (WAS) anaerobic fermentation has drawn much attention^1^,^2^, since sustainable SCFA can be generated in wastewater treatment plants (WWTPs) itself and the waste biomass can be reduced simultaneously. In principle, there are four continuous steps (i.e., solubilization, hydrolysis, acidification, and methanogenesis) involved in the sludge anaerobic fermentation^3^,^4^. SCFA is generated in the acidification step and further consumed in methanogenesis process. SCFA production from WAS anaerobic fermentation is closely relevant to the following two parts: the operational condition of fermentation reactor and inherent characteristics of WAS used.

The operational condition, especially pretreatment of sludge to accelerate sludge hydrolysis rate is an effective approach to promote SCFA production from sludge^5^,^6^. Several pretreatment methods have been documented in the literature so far^5^,^6^,^7^,^8^,^9^,^10^. For example, Zhang and his co-workers reported ultrasonic treatment at a frequency of 0.5 W/mL for half an hour could effectively cause sludge breakdown and bacterial cells disruption^7^. Yuan *et al*. reported that pH at 10 significantly increased soluble protein and carbohydrate content, thereby providing more available substances for SCFA generation^6^. It was found that 48% of chemical oxygen demand (COD) solubilization was achieved when WAS was pretreated with thermal at 190 °C for 1 h^8^. In addition, Yang *et al*. demonstrated that WAS hydrolysis was efficiently enhanced by additional enzymes^9^. Although sludge solubilization and SCFA production could be greatly enhanced by those approaches mentioned, intensive energy inputs or large chemical (biological) consumptions are required in real applications with substantial economic and environmental costs^10^.

Recently, free nitrous acid (FNA), serving as a pretreatment method to stimulate sludge hydrolysis has drawn much attention. FNA, at parts per million levels, acts as a strong biocidal agent for a broad range of microorganisms in WAS^11^,^12^,^13^. Wang *et al*. reported that sludge disintegration was significantly bio-augmented after the pretreatment of FNA at 2.13 mg/L for 24 h^14^. Li *et al*. confirmed that FNA pretreatment improved the SCFA production from WAS anaerobic fermentation^13^. In a recent study, Zhao and his coworkers revealed that the underlying mechanism of how FNA pretreatment accelerated the disruption of extracellular polymeric substances (EPS) and cell envelop^5^. Moreover, FNA was integrated with heat or alkaline conditions to enhance methane or SCFA yield, respectively^5^,^15^. FNA pretreatment as a novel cell disruption technique has many advantages over other methods, such as less intensive energy inputs and chemical consumptions since FNA is a renewable and cost-effective chemical that could be obtained *in situ* by partial nitrification of the anaerobic WAS digestion liquor^14^. Thus, FNA pretreatment seems to be one promising approach to enhance energy recovery from WAS from the aspects of operation control.

Sodium dodecylbenzene sulfonate (SDBS), a micro toxicity and low cost anionic surfactant, has been extensively used as detergents, foaming agent, and dispersants in wastewater treatment plants^16^. The non-degradable SDBS could be accumulated in water body and subsequently transported into WAS with significant amounts^17^. As a result, the characteristics of sludge may be altered significantly, thereby affecting the fermentation process. In the literature, the presence of SDBS was demonstrated to remarkably enhance SCFA accumulation from WAS anaerobic fermentation, as SDBS accelerated WAS solubilization and improved the acidification of hydrolyzed products^16^,^17^.

Although some efforts have been paid to the fermentation process affected by either FNA pretreatment or SDBS levels in sludge, all these investigations were one-dimensional studies. It is possible that some synergetic effects may be caused when the FNA pretreatment and sludge SDBS effect are combined. So far, the combined effect of FNA pretreatment and SDBS on WAS anaerobic fermentation has never been investigated.

The purpose of the paper is to investigate the combined effect of FNA pretreatment and SDBS on SCFA production from WAS anaerobic fermentation. Firstly, the effect of FNA concentration on SCFA production in the presence of SDBS was assessed in a series of batch fermentation reactors. As the combined system was more effective in the SCFA production than FNA pretreatment or SDBS effect alone, the mechanisms of how the combined system produced greater SCFA production than their individuals were investigated by the assays of four steps involved in WAS anaerobic fermentation.

## Results and Discussion

### Effect of the combined FNA pretreatment and SDBS treatment on SCFA production from WAS

**Figure 1:**
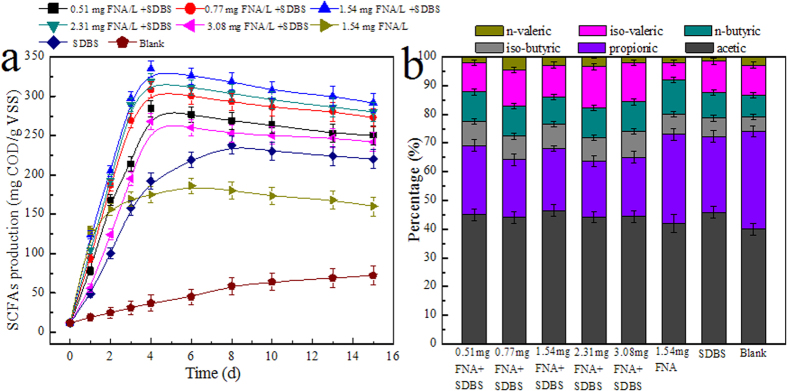
Effect of FNA concentration on SCFA generation from WAS anaerobic fermentation with the combined FNA and SDBS treatment (**a**) and the fraction of individual SCFA under the optimal condition (**b**). The dosage of SDBS was 0.02 g/g DS. Results are the averages and their standard deviations of triplicate measurements.

FNA concentration is considered as an important factor to assess the combined effect of FNA pretreatment and SDBS on SCFA production from WAS anaerobic fermentation. As shown in Fig. 1a, SCFA production was significantly increased in the sole 1.54 mg FNA/L and the sole SDBS reactors, and the maximal SCFA production of 186.5 mg COD/g volatile suspended solids (VSS) and 238.2 mg COD/g VSS were achieved on 6 d and 8 d, respectively. Apparently, the sole pretreatment of FNA and the sole addition of SDBS enhanced the SCFA production in comparison to the blank, which was in agreement with the results reported in the literature^5^,^13^,^17^. However, the combined FNA pretreatment and SDBS addition further improved the SCFA production from WAS fermentation. It should be noted that the SCFA production showed a positive correlation with FNA concentration in a range of 0.51–1.54 mg FNA/L, whereas a negative correlation was observed when FNA concentration further increased from 1.54 mg FNA/L to 3.08 mg FNA/L in the combined treatment. The highest SCFA in the combined FNA pretreatment and SDBS addition was 345.5 mg COD/g VSS, which was nearly 1.79-, 1.41- and 4.8-fold of that in the sole FNA reactor, the sole SDBS reactor, and the blank.

Additionally, the required fermentation time of maximal SCFA production in the combined reactor was only 4 d, whereas 6, 8, and 15 d, were required in the sole FNA reactor, the sole SDBS reactor, and the blank, respectively. It should be noted that the SCFA produced from the FNA pretreated sludge and SDBS added sludge alone at time of 4 d was respectively 175 and 192 mg COD/g VSS, which was approximately half of that produced from the combined reactor. Thus, it can be concluded that the SCFA production was greatly improved with FNA pretreatment in the presence of SDBS.

As shown in Table S1 and Table S2 (Supporting Information), the concentrations of both NO_2_^−^-N and NO_3_^−^-N decreased with fermentation time, which suggested that biological denitrification was observed in all nitrite added reactors. For instance, the NO_2_^−^-N concentration in the 1.54 mg FNA/L + SDBS reactor decreased from 600 mg/L on 1 d to 185.6 mg/L on 6 d, and further to 5.2 mg/L on 15 d. Similar observations were also made in other nitrite added reactors. The low NO_2_^−^-N concentration contained would not pose a negative impact in the following biological utilization.

In order to further clarify the degradation of SDBS in the combined system, the mass balance of SDBS in the aqueous and sludge phases was performed. As displayed in Table S3 (Supporting Information), no significant variation was determined in the sum of aqueous and sludge phases during the 15 d fermentation (p > 0.05), which indicated that SDBS was not degraded during the fermentation process. The initial SDBS content in system was 269.82 mg, however, the final SDBS content was 267.69 mg after 15 d. These results indicate that the SCFA produced in this study were not from the decomposition of SDBS, coinciding with Jiang *et al*. who also reported that SDBS was not degraded in an anaerobic system^17^.

The composition of SCFA is an important parameter affecting the down-stream application^2^. The composition of SCFA can be influenced by many factors such as pH, temperature and sludge retention time (SRT) during sludge fermentation^2^,^18^. It was observed that acetic acid and propionic acid were the dominant products in all reactors, and both two accounted for 75–83% of total SCFA (Fig. 1b). The order of individual SCFA content was acetic > propionic > iso-valeric > n-butyric >iso-butyric > n-valeric in all reactors. Acetic acid and propionic acid could be generated from the degradation of protein and polysaccharide in extracellular polymers (EPS)^19^, which are the dominant substances of sludge. Further analysis revealed that under the optimal conditions there was no significant difference in the percentage of individual SCFA (p > 0.05).

### Mechanisms of how the combined FNA pretreatment and SDBS presence enhanced the SCFA production

#### Effect of the combined FNA pretreatment and SDBS presence on the solubilization of sludge organic matter

Generally, the organic matter in WAS is usually in the particulate form, and the solubilization of sludge organic matter is considered as the first step of the fermentation process^16^,^20^,^21^. Protein and polysaccharide are the main constituents of WAS, and they are also considered as the primary substrates for SCFA production^22^. Thus, the solubilization of sludge particulate organic matter can be measured by the change of soluble protein and polysaccharide in the fermentation liquor. As no extra inoculums were introduced in this part, thus the solubilization substrates (e.g., soluble protein and soluble carbohydrate) were from the fermentation substrates. As shown in Table 1, both the soluble protein and polysaccharide in the combined reactor were greater than those in the individual reactor, which suggested that the combined FNA pretreatment with SDBS presence provided more soluble substrates for SCFA production. In addition, VSS reduction is also relevant to sludge solubilization, and higher VSS reduction was determined under the combined FNA and SDBS condition. For example, 35.5% VSS reduction was achieved in the combined reactor on 3 d, whereas the corresponding data were 24.6%, 29.5% and 13.7% in the sole FNA reactor, the sole SDBS reactor, and the blank, respectively. Those results indicated that the combined FNA pretreatment and SDBS presence improved sludge solubilization.

It was documented that protein and polysaccharide are the main components of sludge EPS, and they are also the key components of microbial cells in the activated sludge system^6^,^23^. Either FNA or SDBS is reported to have ability to promote the solubilization of sludge protein and polysaccharide^5^,^17^. When the FNA pretreatment was integrated with SDBS, the sludge solubilization was enhanced.

#### Effect of the combined FNA pretreatment and SDBS presence on the hydrolysis of soluble protein and polysaccharide

**Figure 2:**
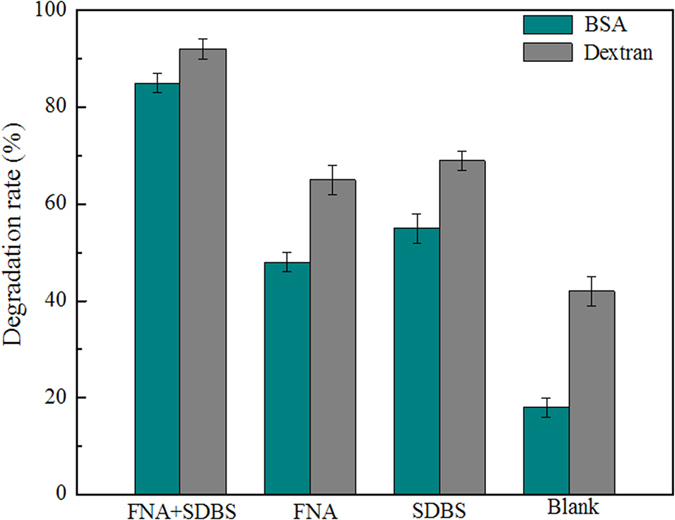
Effect of different treatments on BSA and dextran degradation on 3 d. The FNA concentration and SDBS dosage were 1.54 mg FNA/L and 0.276 g SDBS/L, respectively. Results are the averages and their standard deviations of triplicate measurements.

Effect of the combined FNA pretreatment and SDBS presence on the hydrolysis of solubilized organic matter was investigated with synthetic wastewater containing either bovine serum albumin (BSA) or dextran. As shown in Fig. 2, the degradation rate of BSA (dextran) on 3 d in the combined reactor was 85% (92%), whereas the corresponding data in the sole FNA reactor, the sole SDBS reactor, and the blank was 48% (65%), 55% (69%), and 18% (42%), respectively. Although partial BSA and dextran might be adsorbed by fermentation sludge, from the great difference of degradation rate shown in Fig. 2, it can be concluded that the degradation rate of BSA and dextran in the combined reactor was greater than those in their individuals.

#### Effect of the combined FNA pretreatment and SDBS presence on the acidification of hydrolyzed products

The hydrolyzed products of protein and polysaccharide, such as amino acid and monosaccharide, could be further converted to SCFA during the following acidification step. Synthetic wastewater containing L-alanine (a model amino compound) and glucose (a model monosaccharide compound) was used to investigate the effect of the combined FNA pretreatment and SDBS presence on the acidification step, and the results are summarized in Table 2. The utilization efficiencies of L-alanine and glucose in the combined reactor were greater than those of their individuals and the blank. For example, the utilization efficiency of L-alanine (glucose) in the FNA + SDBS reactor was 86.9% (90.8%) on 3 d, whereas the corresponding data in the sole FNA reactor and the sole SDBS reactor were 69.2% (77.4%) and 75.2% (79.2%), respectively. It was also reported that both FNA and SDBS could enhance acidification of hydrolyzed products in the literature^5^,^16^,^24^. It should be emphasized that when the FNA pretreatment and SDBS addition were combined, the utilization efficiency of L-alanine (glucose) was significantly improved, as compared with their individual (p < 0.05). Apparently, the combined FNA pretreatment and SDBS presence improved acidification of hydrolyzed products.

#### Effect of the combined FNA pretreatment and SDBS presence on the methane generation during sludge fermentation

**Figure 3:**
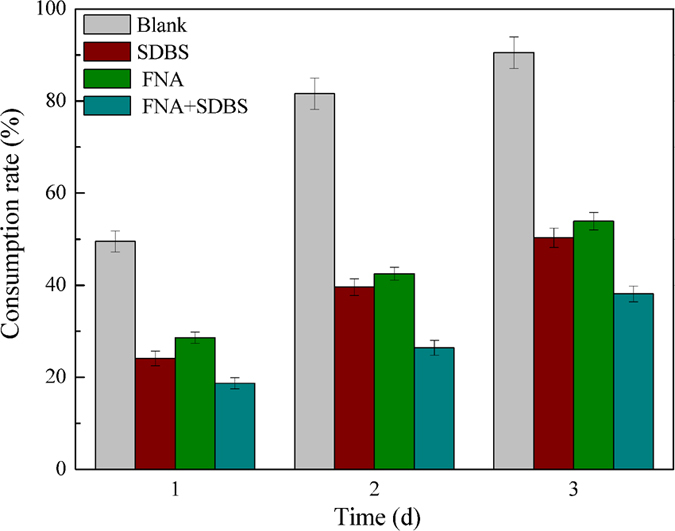
Effect of different treatment conditions on the consumption rate of acetate with fermentation time. The FNA concentration and SDBS dosage were 1.54 mg FNA/L and 0.276 g SDBS/L, respectively. Results are the averages and their standard deviations of triplicate measurements.

It is known that SCFA generated in acidification step can be further consumed in methanogenesis. The effect of the combined treatment on methanogenesis was therefore investigated, and the results are illustrated in Fig. 3. In general, acetate consumption rate was increased with fermentation time in all reactors. When SDBS or FNA was in the presence, the consumption of acetate was seriously inhibited, for example, the consumption rate of acetate in blank was 90.5% at the fermentation time of 3 d, the corresponding data in the SDBS and FNA treated reactor were 50.3% and 53.9%, respectively. However, when SDBS and FNA were combined, the consumption rate of acetate was further inhibited. The degradation rate of acetate was only 38.1% on 3 d, which suggested the process of methanogenesis was seriously inhibited.

It was reported that the presence of surfactants could pose a negative effect on methanogens by disrupting cell membrane during anaerobic fermentation in the literature^20^,^25^. Furthermore, the addition of NO_2_^−^-N could break the strict anaerobic condition, which subsequently decreased the activities of obligatory anaerobic methanogens. According to the results obtained in this study, a synergy inhibitory effect on methanogenesis process when FNA and SDBS treatment are combined.

#### Is denitrification happened in WAS anaerobic fermentation cost-effective?

As is known that denitrification of nitrite would consume a lot of carbon source, this it is necessary to the cost-effect of the FNA-based method to improve SCFA from WAS.

Firstly, it should be emphasized that in real situations the chemical, nitrite used in the fermentation systems can be produced as a byproduct of wastewater treatment through nitritation of the anaerobic digestion liquor. Anaerobic digestion liquor generated in the methane production reactor typically contains 0.8–1.5 g ammonia-nitrogen/L, which can guarantee the level of nitrite required. This means the FNA-based method proposed in this study does not increased extra chemical addition. Then, the maximum SCFA generation from WAS fermentation without treatment was 72 mg COD/g VSS in this work. Whereas, SCFA generation from WAS with 600 mg NO_2_^−^-N/L (pH = 6.0 for 2 d) was 186.5 mg COD/g VSS, which was much higher than that in blank. This means the SCFA production from WAS can be greatly promoted by FNA treatment, and the generated SCFA can be introduced into the influent of wastewater treatment plant to solve the shortage of the carbon source. In addition, denitrification of nitrite will consume carbon sources, and the consumption of carbon resource for nitrite (600 mg/L) denitrification is approximate 1156 mg COD/L according to the literature^13^, which is much less than that produced by FNA treatment (1156 versus 1887 mg COD/L). This comparison clearly shows that FNA-based treatment can improve SCFA accumulation even though lots of carbon resource are consumed for denitrification of nitrite. Finally, if the anaerobic digestion liquor with NO_2_^−^-N is directly introduced into the influent of wastewater treatment plant, consumption of carbon sources will also occur via denitrification of nitrite, which will lower the inherent carbon source content of the influent of wastewater treatment plant. Thus, denitrification happened in WAS anaerobic fermentation is cost-effective for the whole WWTPs.

#### Effects of the combined FNA pretreatment and SDBS presence on fermentation by-products: NH_4_^+^-N and PO_4_^3−^-P concentration

**Figure 4:**
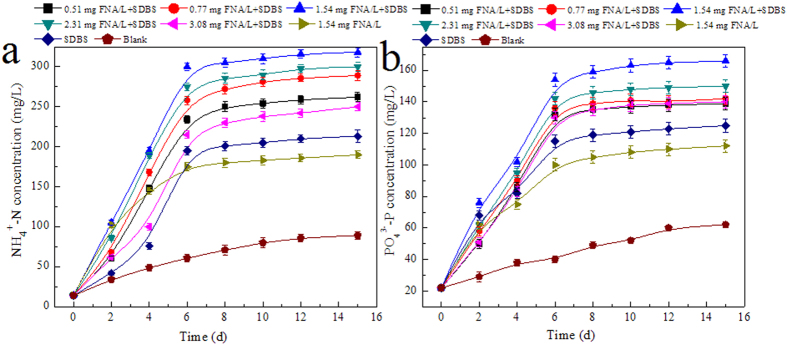
Effect of FNA concentration on the variation of NH_4_^+^-N (**a**) and PO_4_^3−^-P (**b**) during WAS anaerobic fermentation. Error bars represent standard deviations of triplicate tests.

It was reported that both soluble NH_4_^+^-N and PO_4_^3−^-P were largely released into the aqueous phase during WAS fermentation^17^,^26^. Unlike the release of protein and polysaccharide that could benefit carbon sources in wastewater treatment processes, the release of nutrients (e.g., soluble NH_4_^+^-N and PO_4_^3−^-P) from the sludge phase to the liquid phase would increase nutrient discharge loadings^27^. As shown in Fig. 4, the soluble NH_4_^+^-N and PO_4_^3−^-P concentrations increased with the fermentation time regardless of the treatment conditions. However, the soluble NH_4_^+^-N and PO_4_^3−^-P concentrations in the combined reactors were higher than those in their individuals. For instance, the NH_4_^+^-N and PO_4_^3−^-P concentrations were 300 mg/L and 154 mg/L on 6 d in the 1.54 mg FNA/L + SDBS reactor, respectively. However, the corresponding data were 175 mg/L and 102 mg/L in the sole 1.54 mg FNA/L treated reactor and 196 mg/L and 112 mg/L in the sole SDBS presence reactor. The level of NH_4_^+^-N release was in agreement with the degradation of protein. NH_4_^+^-N is mainly produced by biological degradation of nitrogenous substrates (mainly sludge protein in this study), which is hydrolyzed to amino acids and further converted to soluble NH_4_^+^-N. The higher soluble NH_4_^+^-N concentration achieved in the combined reactor suggested the higher sludge degradation efficiency. The NH_4_^+^-N and PO_4_^3−^-P rich fermentation liquid should be introduced into N and P recovery and separation system before SCFA application^4^.

## Conclusion

The effect of the combined FNA pretreatment and SDBS presence on SCFA production was investigated. Results showed that a synergy effect on SCFA production from WAS anaerobic fermentation occurred when the FNA pretreatment and SDBS presence were combined. The highest SCFA yield of 334.5 mg COD/g VSS was achieved at 1.54 mg FNA/L pretreatment for 2 d with the presence of SDBS, which was much higher than those from their individuals. Mechanism investigations revealed that the combination of FNA pretreatment and SDBS presence accelerated the solubilization, hydrolysis, and acidification steps, but inhibited the methanogenesis step.

## Materials and Methods

### WAS source

WAS used in this study was withdrawn from the secondary sedimentation tank of a municipal WWTP (treatment capacity: 500,000 m^3^/d) in Changsha, China. The plant is operated as anaerobic-anoxic-oxic process for biological nutrient removal. The SRT is about 15 d. The collected sludge was gravitational sedimentation for about 24 h by settling at 4 °C prior to use, and the main characteristics of WAS after concentration are as follows: total suspended solids (TSS) 13800 ± 208 mg/L, VSS 10120 ± 145 mg/L, total chemical oxygen demand (TCOD) 14150 ± 220 mg/L, soluble COD 224 ± 20 mg/L, total polysaccharide 1331 ± 31 mg COD/L, total protein 8019 ± 105 mg COD/L, pH 6.8 ± 0.1. Error bar means standard deviations of triplicate measurements. As shown above, protein and polysaccharide are the two predominant organic compounds of WAS.

### Effect of FNA concentration on the SCFA production in the presence of SDBS

This investigation was carried out in eight identical fermentation reactors, which were made of plexiglass and each had a working volume of 1.0 L. The SDBS concentration in this investigation was selected as 0.02 g/g dry sludge (DS) according to the literature^16^,^17^. Sludge pretreatment with several FNA concentrations (0.51, 0.77, 1.54, 2.31 and 3.08 mg FNA/L) was conducted to gain a comprehensive understanding of how the FNA pretreatment affected SCFA generation in the presence of SDBS. Each batch test was conducted in fermentation reactors mixed with stirrers at a speed of 100 rpm (revolutions per minute) in a 20 ± 1 °C temperature-controlled room. A nitrite stock solution (2.0 M) was first added into different reactors with different volumes to obtain the pre-designed nitrite concentration i.e., 200, 300, 600, 900 and 1350 mg NO_2_^−^-N/L. Then, pH in all reactors was maintained at 6.0 ± 0.1 by adding 3.0 M HCl or 3.0 M NaOH through a programmable logic controller during pretreatment phase. Afterwards, the headspace of the reactor was purged with nitrogen for 15 min to provide anaerobic condition. The temperature, pH and nitrite concentration applied gave rise to FNA concentrations of 0.51, 0.77, 1.54, 2.31 and 3.08 mg FNA/L, respectively, which were determined by the formula FNA = S_NO2_^−^-_N_/(K_a_ × 10 ^pH^) and the value of K_a_ was determined by the formula K_a_ = e^(−2300/(T+273))^ for a given temperature T (°C)^28^. The FNA pretreatment time was maintained for 2 d according to our previous publications^5^,^13^. In addition, another 3 reactors were also conducted, two of them were treated respectively with only 1.54 mg FNA/L and 0.02 g/g DS SDBS according to the preliminary results of this study, and the third one served as a blank (uncontrolled). In this batch test, no extra inoculums were added, and the raw sludge was employed for both fermentation substrates and inoculums. Other treatment conditions were the same as the above description.

### Mechanism investigations for SCFA production stimulated by the combined FNA pretreatment and SDBS

In order to dig out the underlying mechanisms of the enhanced SCFA production with the combined FNA pretreatment and SDBS, the effects of the combined treatment on the four steps involved in WAS anaerobic fermentation were studied. Since protein and polysaccharide are the main substrates in WAS, the variations of soluble protein and polysaccharide in the fermentation liquor could be applied to indicate the effect of the combined treatment on sludge solubilization.

The effect of the combined treatment on hydrolysis of solubilized organic matters was investigated with synthetic wastewater containing bovine serum albumin (BSA, average molecular weight Mw 67000, a model protein compound) and dextran (Mw 23,800, a model polysaccharide compound) in four anaerobic reactors (working volume 1L). The BSA and dextran concentrations in each reactor were 5.3 g/L and 1.2 g/L, respectively. Those concentrations of model substrates are similar to those in the raw sludge. After the addition of BSA or dextran, 100 mL of raw WAS was also immediately added into each reactor serving as inoculums. The first reactor was treated with the FNA pretreatment at the presence of SDBS, the second and third ones were respectively treated with FNA and SDBS alone, and the last one served as blank without neither FNA pretreatment nor the addition of SDBS. The initial FNA concentration was maintained at 1.54 mg FNA/L and the SDBS dosage was controlled at 0.276 g SDBS/L, which was similar to that in the reactors for SCFA generation. The other operation conditions were the same as described above. Another four anaerobic fermentation reactors fed with 100 mL of raw sludge and 900 mL of tap waster each were conducted to evaluate the impact of treatment conditions on inoculums. By measurement of the remainder BSA and dextran in fermentation liquor, the hydrolysis rate could be obtained. The hydrolysis efficiencies of model substrates can be expressed by the following equation.


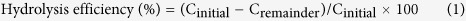


where, the C _initial_ and C _remainder_ are the concentrations of BSA and dextran in reactor before and after different treatment, respectively. The data reported were the net results that the data obtained in reactors containing inoculums and substrates subtracted the ones in reactors containing sole inoculums under the same conditions.

The investigation of the combined treatment influence on the acidification step was similar to that in hydrolysis step expect that the model substrates in synthetic wastewater were replaced with L-alanine (a model amino acid compound) and glucose (a model monosaccharide compound). The concentrations of L-alanine and glucose in synthetic wastewater were 2.0 g/L and 0.5 g/L, respectively. Other operational conditions were the same as those described above. Also, two reproductive reactors with 100 mL of raw sludge and 900 mL of tap water were carried out to assess the influence of treatment condition on inoculums. The simulation experiment has been widely used in previous studies^5^,^6^,^17^.

The investigation of the combined treatment influence on methanogenesis of acidified compounds was conducted in anaerobic reactors with synthetic wastewater containing sodium acetate (NaAc) (0.5 g/L). Other chemical composition of synthetic wastewater was the same as those described in the literature^20^. 100 mL of raw WAS serving as inoculums was added into each reactor. To avoid the influence of pH variation on methanogenesis, pH was controlled constantly at 7.0 by adding 3.0 M HCl or 3.0 M NaOH. Then, the first reactor was treated with 6000 mg NO_2_^−^-N/L and 0.276 g SDBS/L (the SDBS content was similar to that in the reactor for SCFA generation), and the second and the third reactors were either received 6000 mg NO_2_^−^-N/L or fermented in the addition of 0.276 g SDBS/L. The last one served as the blank. The temperature was maintained at 20 ± 1 °C, and the temperature, pH and nitrite concentration applied gave rise to FNA concentration of 1.54 mg FNA/L. All other operational conditions were the same as described above. Thus, consumption rate of acetate could be applied to assess the effect of the combined FNA pretreatment and SDBS on methanogenesis process can be expressed by the consumption rate of acetate. In addition, another four anaerobic reactors fed with 100 mL of raw sludge and 900 mL of tap water each were performed to assess the effect of treatment conditions on inoculums. The data reported in this study were the net results that the data obtained in reactors containing inoculums and substrates subtracted the ones in reactors containing sole inoculums under the same conditions.

## Analytical Methods

The analyses of TSS, VSS, NH_4_^+^-N, PO_4_^3−^-P, and soluble COD were performed according to Standard Methods^29^. Soluble protein and carbohydrate were determined by the Lowry-Folin method with BSA as the standard and the phenol-sulfuric method with glucose as the standard, respectively^5^,^30^. The measurement of BSA, dextran, L-alanine, glucose, SCFA, SDBS and gas component were the same as described in the literatures^5^,^6^,^17^.

### Statistical Analysis

All measurements in this study were conducted in triplicate. An analysis of variance was used to evaluate the significance of results, and p < 0.05 was considered to be statistically significant.

## Additional Information

**How to cite this article**: Zhao, J. *et al*. Combined Effect of Free Nitrous Acid Pretreatment and Sodium Dodecylbenzene Sulfonate on Short-Chain Fatty Acid Production from Waste Activated Sludge. *Sci. Rep.* **6**, 21622; doi: 10.1038/srep21622 (2016).

## Supplementary Material

Supplementary Information

## Figures and Tables

**Table 1:** Variations of soluble protein, polysaccharide and VSS reduction with time in different reactors^a^.

	Time (d)	Soluble protein (mg/L)	Soluble polysaccharide (mg/L)	VSS reduction (%)
FNA + SDBS^b^	1	758 ± 21	108 ± 5	12.3 ± 0.5
	2	1254 ± 36	215 ± 10	26.9 ± 0.9
	3	1545 ± 38	304 ± 14	35.5 ± 1.2
FNA	1	325 ± 15	58 ± 3	9.5 ± 0.4
	2	658 ± 19	113 ± 5	18.6 ± 0.8
	3	854 ± 24	165 ± 7	24.6 ± 1.2
SDBS	1	584 ± 18	75 ± 4	10.3 ± 0.6
	2	859 ± 25	148 ± 6	21.9 ± 1.1
	3	1025 ± 31	196 ± 8	29.5 ± 1.6
Blank	1	185 ± 10	48 ± 2	5.4 ± 0.3
	2	247 ± 12	77 ± 3	10.2 ± 1.2
	3	318 ± 14	95 ± 4	13.7 ± 1.3

^a^The data are the averages and their standard deviations in triplicate tests.

^b^The FNA concentration was 1.54 mg FNA/L, SDBS dosage was 0.02 g/g DS.

**Table 2:** Effect of sole FNA, sole SDBS, and FNA + SDBS on degradation rate of model compounds with time^a^.

Treatment	Time (d)	L-alanine degradation (%)	Glucose degradation (%)
1.54 mg/L FNA	1	41.1 ± 0.4	30.3 ± 0.4
	2	62.3 ± 0.6	69.7 ± 0.5
	3	69.2 ± 0.7	77.4 ± 1.1
SDBS^b^	1	43.6 ± 0.5	35.1 ± 0.8
	2	68.9 ± 0.6	68.7 ± 0.7
	3	75.2 ± 0.5	79.2 ± 0.6
1.54 mg/L FNA + SDBS	1	49.6 ± 0.6	40.5 ± 0.4
	2	78.7 ± 0.7	76.9 ± 0.5
	3	86.9 ± 0.5	90.8 ± 0.7

^a^The data are the averages and their standard deviations in triplicate tests.

^b^The SDBS dosage was 0.276 g SDBS/L.
