# Supplementary material for: Combined Effect of Free Nitrous Acid Pretreatment and Sodium Dodecylbenzene Sulfonate on Short-Chain Fatty Acid Production from Waste Activated Sludge
Source: Sci Rep. 2016 Feb 12;6:21622. doi: 10.1038/srep21622 (PMC4751509; doi:10.1038/srep21622)
Supplement: Supplementary Information [file srep21622-s1.pdf]

Supporting Information

Combined Effect of Free Nitrous Acid Pretreatment and Sodium Dodecylbenzene Sulfonate on Short-Chain Fatty Acid Production from Waste Activated Sludge

Jianwei Zhao [1,2,†], Yiwen Liu[3,†], Bingjie Ni[3], Qilin Wang[3], Dongbo Wang [1,2,3*] Qi Yang [1,2], Yingjie Sun[4], Guangming Zeng [1,2], Xiaoming Li [1,2*]

[1] College of Environmental Science and Engineering, Hunan University, Changsha 410082, P.R. China

[2] Key Laboratory of Environmental Biology and Pollution Control (Hunan University), Ministry of Education, Changsha 410082, P.R. China

[3] Advanced Water Management Centre, The University of Queensland, QLD 4072, Australia

[4] School of Environment and Municipal Engineering, Qingdao Technological University, Qingdao 266033, P.R. China

* Corresponding author. Tel.: +86 731 88823967; fax: +86 731 88822829.
E-mail addresses: w.dongbo@yahoo.com (D. Wang); xmli@hnu.edu.cn (X. Li).

Table S1. Variation of NO_2^--N in the combined FNA and SDBS treatment reactors with time.[a]

	Treatment time (d)				
Reactor	0	3	6	9	15
0.51 mg FNA/L+SDBS	200.0 ± 2.1	85.6 ± 1.5	27.2 ± 1.1	5.3 ± 0.1	0.5± 0.1
0.77 mg FNA/L+SDBS	300.0 ± 2.8	192.7 ± 2.2	91.5 ± 2.2	21.6 ± 0.2	2.5 ± 0.1
1.54 mg FNA/L +SDBS	600.0 ± 4.2	428.9 ± 3.6	185.6± 5.6	37.9 ± 0.6	5.2 ± 0.1
2.31 mg FNA/L+SDBS	900.0 ± 4.3	723.9 ± 3.9	425.1 ± 6.7	156.9 ± 3.2	21.6 ± 0.2
3.08 mg FNA/L+SDBS	1350.0 ± 4.5	821.6 ± 5.4	438.9 ± 6.2	185.4 ± 4.1	26.8 ± 0.2

[a] Error bars represent standard deviations of triplicate measurements, and NO_2^--N concentrations are expressed in mg/L.

Table S2. Variation of NO_3^--N in the combined FNA and SDBS treatment reactors with time.[a]

	Treatment time (d)				
Reactor	0	3	6	9	15
0.51 mg FNA/L+SDBS	24.3 ± 2.1	13.6 ± 1.5	6.7 ± 0.4	2.4 ± 0.3	0.1 ± 0.05
0.77 mg FNA/L+SDBS	24.3 ± 2.1	15.4 ± 1.7	7.8 ± 0.6	3.2 ± 0.4	0.3 ± 0.1
1.54 mg FNA/L +SDBS	24.3 ± 2.1	16.7 ± 2.1	8.2 ± 0.5	4.6 ± 0.4	0.5 ± 0.1
2.31 mg FNA/L+SDBS	24.3 ± 2.1	18.2 ± 1.8	9.5 ± 0.8	5.1 ± 0.6	0.6 ± 0.1
3.08 mg FNA/L+SDBS	24.3 ± 2.1	18.9 ± 2.4	10.2 ± 1.4	5.5 ± 0.7	0.8 ± 0.1

[a] Error bars represent standard deviations of triplicate measurements, and NO_3^--N concentrations are expressed in mg/L.

Table S3. Variations of SDBS with fermentation time in the fermentation system [a]

Fermentation time (d)	1	3	5	7	9	12	15
SDBS in aqueous phase (mg)	52.39	52.95	53.12	53.24	53.31	53.38	53.40
SDBS in sludge phase (mg)	217.43	216.63	216.06	215.83	215.20	214.89	214.29
Total SDBS in system (mg)	269.82	269.58	269.18	269.07	268.51	268.27	267.69

[a] Error bars represent standard deviations of triplicate measurements.
